# Associations of *TRAF2* (rs867186), *TAB2* (rs237025), *IKBKB* (rs13278372) Polymorphisms and *TRAF2*, *TAB2*, *IKBKB* Protein Levels with Clinical and Morphological Features of Pituitary Adenomas

**DOI:** 10.3390/cancers16142509

**Published:** 2024-07-10

**Authors:** Balys Remigijus Zaliunas, Greta Gedvilaite-Vaicechauskiene, Loresa Kriauciuniene, Arimantas Tamasauskas, Rasa Liutkeviciene

**Affiliations:** 1 Medical Faculty, Lithuanian University of Health Sciences, Medical Academy, 44307 Kaunas, Lithuania; balys.remigijus.zaliunas@stud.lsmu.lt; 2 Neuroscience Institute, Lithuanian University of Health Sciences, Medical Academy, 44307 Kaunas, Lithuania; loresa.kriauciuniene@lsmuni.lt (L.K.); rasa.liutkeviciene@lsmuni.lt (R.L.); 3 Department of Neurosurgery, Lithuanian University of Health Sciences, Medical Academy, 44307 Kaunas, Lithuania; arimantas.tamasauskas@lsmuni.lt

**Keywords:** pituitary adenoma, gene polymorphisms *TRAF2* (rs867186), *TAB2* (rs237025), *IKBKB* (rs13278372) ELISA, *TRAF2*, *TAB2*, *IKBKB*

## Abstract

**Simple Summary:**

This study investigated the relationship between specific gene polymorphisms (*TRAF2, TAB2, IKBKB*) and protein levels and pituitary adenomas (PAs). The research included 459 participants, divided into a control group and a PA group. The key findings include significant differences in *TRAF2* genotypes between the groups, with the G allele being less common in the PA group. The presence of the G allele and GG genotype were linked to a reduced risk of developing PAs, particularly microadenomas and macroadenomas. These results indicate a protective effect of the *TRAF2* G allele against pituitary tumors.

**Abstract:**

Aim: The aim of this study was to determine associations of *TRAF2* (rs867186), *TAB2* (rs237025), *IKBKB* (rs13278372) gene polymorphisms and *TRAF2*, *TAB2*, *IKBKB* protein levels with clinical and morphological features of pituitary adenomas (PAs). Methods: This case–control study included 459 individuals divided into two groups: a control group (*n* = 320) and a group of individuals with PAs (*n* = 139). DNA from peripheral blood leukocytes was isolated using salt precipitation and column method. Real-time PCR was used for *TRAF2* (rs867186), *TAB2* (rs237025), and *IKBKB* (rs13278372) SNP genotyping, and TRAF2, TAB2, IKBKB protein concentration measurements were performed by immunoenzymatic analysis tests using a commercial ELISA kit according to the manufacturer’s recommendations. The labeling index Ki-67 was determined by immunohistochemical analysis using a monoclonal antibody (clone SP6; Spring Bioscience Corporation). Statistical data analysis was performed using the programs "IMB SPSS Statistics 29.0". Results: We found significant differences in *TRAF2* (rs867186) genotypes (AA, AG, GG) between groups: 79.1%, 17.3%, 3.6% vs. 55.3%, 20.9%, 23.8% (*p* < 0.001). The G allele was less frequent in the PA group than in controls (12.2% vs. 34.2%, *p* < 0.001). The AG and GG genotypes reduced PA occurrence by 1.74-fold and 9.43-fold, respectively, compared to AA (*p* < 0.001). In the dominant model, GG and AG genotypes reduced PA odds by 3.07-fold, while in the recessive model, the GG genotype reduced PA odds by 8.33-fold (*p* < 0.001). Each G allele decreased PA odds by 2.49-fold in the additive model (*p* < 0.001). Microadenomas had significant genotype differences compared to controls: 81.3%, 18.8%, 0.0% vs. 55.3%, 20.9%, 23.8% (*p* < 0.001), with the G allele being less frequent (9.4% vs. 34.2%, *p* < 0.001). In macroadenomas, genotype differences were 78%, 16.5%, 5.5% vs. 55.3%, 20.9%, 23.8% (*p* < 0.001), and the G allele was less common (13.7% vs. 34.2%, *p* < 0.001). The dominant model showed that GG and AG genotypes reduced microadenoma odds by 3.5-fold (*p* = 0.001), and each G allele reduced microadenoma odds by 3.1-fold (*p* < 0.001). For macroadenomas, the GG genotype reduced odds by 6.1-fold in the codominant model (*p* < 0.001) and by 2.9-fold in GG and AG genotypes combined compared to AA (*p* < 0.001). The recessive model indicated the GG genotype reduced macroadenoma odds by 5.3-fold (*p* < 0.001), and each G allele reduced odds by 2.2-fold in the additive model (*p* < 0.001). Conclusions: The *TRAF2* (rs867186) G allele and GG genotype are significantly associated with reduced odds of pituitary adenomas, including both microadenomas and macroadenomas, compared to the AA genotype. These findings suggest a protective role of the G allele against the occurrence of these tumors.

## 1. Introduction

A pituitary adenoma (PA) is a benign intracranial tumor that grows from the adenohypophyseal cells of the anterior part of the post-brain gland and occurs in 10–20% of all cases of individuals in the general population [1]. PAs account for about 15 of all intracranial tumors [2]. Radiological and autopsy studies show that PAs occur in an average of 10% of the general population. Around 99.9% of PAs are microadenomas. Clinically manifest PAs occur in about 1 in 1100 people in the general population. Of these, 48% are macroadenomas [3].

From 2004 to 2016, the standardized incidence rate of PAs was 4.8 per 100,000 inhabitants, with higher rates in women (5.3) than in men (4.3), according to National Cancer Institute data. The incidence increased annually, peaking in 2015 at 5.8 per 100,000. In women, the age distribution showed bimodal peaks at 25–34 and 60–69 years. The survival rates at 3, 5, and 10 years were 94.3%, 91.3%, and 83.1%, respectively [4]. According to the data of the Cancer Registry of the National Cancer Institute, 265 new cases of malignant and 205 benign brain tumors were registered in Lithuania in 2017. To our knowledge, there is no information on the incidence of PAs [5].

Most PAs (65–70%) are characterized by hypersecretion of prolactin, growth hormone, adrenocorticotropic hormone, and thyrotropic hormone. The remainder of PAs (30–35%) are adenomas that do not produce hormones and often cause hypopituitarism [6]. Considering the clinical symptoms, PAs typically cause syndromes related to hormone hypersecretion, such as acromegaly, hyperprolactinemia, hyperthyroidism, and Cushing’s syndrome due to somatotropin, prolactin, thyrotropin, and corticotropin hypersecretion, respectively [7]. About 6–10% of PAs invade the cavernous sinus, potentially causing paralysis of cranial nerves III, IV, and VI [8]. Paralysis of these nerves can result from direct tumor compression, pressure on the cavernous sinus wall, tumor bleeding, or direct infiltration. A PA most commonly affects cranial nerves III and VI, with cranial nerve V being the least affected [9].

The exact etiology and pathogenesis of PAs are still not fully understood. Still, many factors are thought to influence its manifestation, including both the environment and hormonal status, as well as genetic risk factors and their interrelationships [10], so scientists are paying particular attention to the links between immunogenetic factors and PAs in order to improve the diagnosis of PAs, predict the clinical course, and choose the most appropriate treatment tactics [11].

*TRAF2* is a protein that activates many transcription factors, including nuclear factor kappa B (NFκB) and MAP kinase [12]. It has been found that patients with multiple myeloma, hepatocellular carcinoma, and prostate cancer are characterized by increased expression of *TRAF2* [13,14,15,16]. In the mid-1990s, Rothe and his colleagues discovered four proteins—cIAP1, cIAP2, TRAF1, *TRAF2*—that can bind to tumor necrosis factor receptor 2 (TNFR2) [17,18]. It has been found that TRAF1 and *TRAF2* can bind directly to activated TNFR2, but the *TRAF2* protein is required for the binding of cIAP1 and cIAP2 to TNFR2. Like other TRAF family proteins, *TRAF2* is involved in several cell signaling pathways that activate transcription factors, such as nuclear factor kappa B (NFκB) and MAP kinases [13]. The *TRAF2* gene is localized on the long arm of chromosome 9 (9q34.3). The activation of the NFκB system has long been associated with developing tumor processes [19]. Under normal conditions, *TRAF2* protein interacting with TRAF3 and cIAP1/2 leads to ubiquitination of NFκB-inducing kinase (NIK), resulting in proteasomal degradation of NIK and inhibition of activation of the alternative NFκB pathway [13].

*TAB2* is a TGF-beta-activated kinase 1 (MAP3K7)-binding protein 2 that is required for IL-1-induced activation of nuclear factor kappa B (NFκB) and MAPK8/JNK. Increased expression of *TAB2* has been shown to lead to the development of oral squamous cell carcinoma and promote tumor cell proliferation and is associated with a poor prognosis [20]. In addition, there are data showing that the *TAB2* polymorphism (rs237028) leads to a higher risk of epithelial ovarian tumors [14,21]. TGF-beta-activated kinase 1 (MAP3K7)-binding protein 2 (*TAB2*) is known as a MAP3K7/TAK1 activator, which is required for IL-1-induced activation of nuclear factor kappa B (NFκB) and MAPK8/JNK. This protein forms a kinase complex with TRAF6, MAP3K7, and TAB1, thus acting as an adapter that connects MAP3K7 and TRAF6. This protein, together with TAB1 and MAP3K7, is also involved in signaling transduction involving TNFSF11/RANKl, the activating receptor activator of NFκB (TNFRSF11A/RANK), which regulates osteoclast development and function [22]. The gene encoding the *TAB2* protein is localized on the long arm of chromosome 6 (6q25.1). Previous studies have confirmed that *TAB2* is vital in activating the NFκB signaling pathway by binding to polyubiquitin chains [23,24]. Notably, active NFκB promotes the activation of EMT and PI3K-AKT signaling pathways, which are associated with tumor cell metastasis and proliferation [25].

The IKKβ protein belongs to the IKK complex, which consists of the IKKα kinase and the essential modulator of NF-κB (NEMO/IKKγ). The IKKβ protein is responsible for the phosphorylation of the inhibitor (IκB) present in the inhibitor–NF-κB complex, which causes the dissociation of the inhibitor and the activation of NF-κB. Subsequently, phosphorylated IκB undergoes K48 ubiquitination, degrading the proteosome inhibitor [26]. Free NFκB that enters the nucleus activates the transcription of many genes involved in cell cycle control and protection from apoptosis [27]. The IKKβ protein is associated with carcinogenesis. The study by Liao and co-authors showed that cisplatin-resistant squamous tumor cells of the head and neck are characterized by greater invasion into the surrounding tissue and higher IKKβ/NF-κB activity compared to healthy cells.

On the other hand, the IKKβ inhibitor CmpdA significantly reduces tumor cell migration and invasion in vitro and metastasis in vivo. *IKBKB* is the gene encoding the IKKβ protein. Head and neck tumor cells with squamous epithelium have been found to have higher IKKβ activity [28]. In addition, the *IKBKB* polymorphism (rs2272736) is known to be associated with poor prognosis in gastric cancer [29]. *IKBKB* is a gene that encodes a Ser/Thr kinase protein known as IKKβ (inhibitor of NFκB kinase beta subunit). This gene is located on the short arm of chromosome 8 (8p11.21).

As mentioned before, the NF-κB signaling pathway plays a critical role in regulating immune responses, inflammation, and cell proliferation. A key aspect of this pathway involves activating and regulating the IκB kinase (IKK) complex, which subsequently influences various downstream effects. The genes *TRAF2*, *TAB2*, and *IKBKB* are particularly noteworthy in this context due to their specific roles and interactions in the pathway. This study, therefore, examines the relationship between the *TRAF2* (rs867186), *TAB2* (rs237025), and *IKBKB* (rs13278372) gene polymorphisms, *TRAF2*, *TAB2*, and *IKBKB* protein levels, and the Ki-67 labeling index and the clinical and morphological signs of PAs, as well as the relationship between their combinations and the manifestation of PAs.

## 2. Material and Methods

### 2.1. Study Group

The subject of this study is the investigation of the polymorphisms *TRAF2* rs867186, *TAB2* rs237025, and *IKBKB* rs13278372 in patients with pituitary adenoma and control groups. This study included 139 patients with pituitary adenoma: 83 women (59.7%) and 56 men (40.3%). The average age of men with pituitary adenoma was 55.8 years, and the average age of women was 52.6 years. Participants in the control group comprised 320 people: 204 women (63.7%) and 116 men (36.3%). The average age of the men was 65.4 years, and that of the women was 50.3 years.

Patients diagnosed with PAs were recruited from a single specialized endocrinology center. Healthy controls were recruited from the general population through advertisements and health check-up camps, ensuring an age and gender distribution similar to that of the PA group.

The inclusion criteria for the PA group required participants to have a diagnosed and confirmed pituitary adenoma through magnetic resonance imaging (MRI), be in good general health, provide informed consent to participate in this study, be aged 18 years and above, and have no other tumors. The control group consisted of participants who matched the gender and age distribution of the PA group, had no history of pituitary adenoma or other tumors, were in good general health, provided informed consent to participate in this study, and were aged 18 years and above.

The exclusion criteria for the PA group included the presence of other tumors and severe comorbidities that could affect the study outcomes. Similarly, for the control group, individuals with a history of pituitary adenoma or other tumors and those with severe comorbidities that could affect the study outcomes were excluded.

For sample collection, blood samples were collected from patients in the PA group after their initial diagnosis of pituitary adenoma during their first clinic visit following the diagnosis. In the control group, blood samples were collected from healthy control subjects who met the inclusion criteria and visited the clinic for general health check-ups.

### 2.2. DNA Extraction and Genotyping

In this study, DNA was extracted from leukocytes from the venous blood of the study participants. To prevent clot formation, the participant’s blood was stored in vacuum tubes with ethylenediaminetetraacetate (EDTA).

The DNA extraction process involved several steps. First, blood samples were processed to isolate leukocytes. Then, DNA was extracted from the leukocytes using the salting out method. Finally, the quality and concentration of the extracted DNA were assessed using a spectrophotometer. The polymorphisms *TRAF2* rs867186, *TAB2* rs237025, and *IKBKB* rs13278372 were determined by DNA denaturation at 94–96 °C, primer hybridization at 40–60 °C, and elongation at 70–75 °C. Genotyping of the samples was performed using a StepOne Plus RT-PCR amplifier (Applied Biosystems, San Francisco, CA, USA). For each reaction, 1.5 µL of the tested DNA and 8.5 µL of the RT-PCR reaction mixture were used. From the prepared TL-PCR reaction mix for 96 samples, 8.5 µL was poured into all plate wells. Subsequently, 1.5 µL of the DNA mixture was added to 95 wells, and 1.5 µL of sterile water was added to the last well for the negative control. The plate was tightly sealed with a special optical film and placed in a centrifuge to remove air bubbles. The plate was then placed in a real-time thermocycler to determine the polymorphism.

The RT-PCR conditions were as follows: Begin with an initial denaturation step at 95 °C for 10 min. This is followed by 45 cycles, each consisting of denaturation at 92 °C for 15 s and annealing and extension at 60 °C for 60 s. The protocol for determining the polymorphism conditions is shown in Table 1.

### 2.3. Determination of the TRAF2, TAB2 and IKBKB Proteins

To determine the levels of *TRAF2*, *TAB2*, and *IKBKB* proteins in the blood serum of subjects in the PA and control groups, an immunoenzymatic assay was performed using commercial ELISA kits according to the manufacturer’s recommendations. Serum samples were collected from blood, centrifuged to remove cells, and stored at −80 °C until analysis.

Calibration curves were determined using reference solutions with known *TRAF2*, *TAB2*, and *IKBKB* protein concentrations. Each plate well was coated with the corresponding antibodies by the manufacturer. Standard solutions and test samples were added to the wells of the ELISA plates at 100 µL. The plates were covered with a special foil and incubated according to the manufacturer’s recommendations. Detection Reagent A was added to the wells, and incubation was continued according to the manufacturer’s recommendations. After incubation, each well was washed three times with a wash buffer. After the first wash, Detection Reagent B was added to each well, and incubation was performed after covering the wells according to the manufacturer’s recommendations. After incubation, the wells were washed 5 times with wash buffer. After washing, 90 µL of the substrate solution was added to each well, incubated in the dark, and covered according to the manufacturer’s recommendations. After incubation, a stop reagent was added to each well, and a color change from blue to yellow was observed. The absorbance was measured using a microplate reader at the appropriate wavelengths. The concentration of the samples was determined using a curve of reference substances.

### 2.4. Determination of the Ki-67 Labeling Index

The Ki-67 labeling index was determined by immunohistochemical analysis using a monoclonal antibody (clone SP6; Spring Bioscience Corporation, Pleasanton, CA, USA). This index indicates the positive percentage of stained tumor cells. A qualified pathologist evaluated the Ki-67 index at the LSMU Pathological Anatomy Clinic. Protein biomarkers were analyzed according to the protocol for immunohistochemical analysis of paraffin sections using the “Ventana BenchMark XT” staining procedure from “Ventana Medical Systems” Tucson, AZ, USA. The paraffin sections were deparaffinized with a “Ventana” reagent; then, the antigenic epitopes were reconstituted with the “Ventana” cell conditioning solution (pH 8.4) for 60 min at a temperature of 100 °C. The monoclonal antibodies were applied to the sections for 32 min at 37 °C and analyzed using the “Ventana iVIEW DAB Detection Kit”. The immunohistochemical reaction was completed by contrasting the sections with Gill’s hematoxylin solution, staining with blue reagent from a buffered aqueous lithium carbonate solution, and coverslipping.

### 2.5. Statistical Data Analysis

The statistical data analysis was carried out using the statistical program package “Statistical Package for the Social Sciences, Version 29.0 for Windows” (SPSS for Windows, Version 29.0, USA). The hypothesis about the normal difference in the values of the measured characteristics was tested by applying the Kolmogorov–Smirnov and Shapiro–Wilks tests. If the subjects’ characteristics did not fulfill the criteria of a normal distribution, the following descriptive statistical characteristics were applied: median, interquartile range (IQR), average rank *TRAF2* rs867186, *TAB2* rs237025, *IKBKB* rs13278372. The χ^2^ test was used to compare the homogeneity of the distribution of single-nucleotide polymorphisms. After a binary logistic regression analysis was performed, the odds ratio (OR) for the occurrence of the disease was estimated, taking into account inheritance patterns and genotype combinations, with the OR expressed with a 95% confidence interval (CI). The Akaike information criterion (AIC) was used to select the best inheritance model, with the lowest value indicating the best-fitting model. To compare the results in different groups when the data distribution was not normal, the non-parametric Mano–Witnis U analysis method was used. A significance level of 0.05 was chosen to test the statistical hypotheses. A statistically significant difference was determined if the *p*-value was less than 0.05 (*p* < 0.05).

## 3. Results

This case–control study comprised 459 subjects divided into two groups: a control group (*n* = 320) and a group of people with pituitary adenoma (*n* = 139). After the study groups were formed, genotyping of the *TRAF2* rs867186, *TAB2* rs237025, and *IKBKB* rs13278372 polymorphisms was performed. The PA group consisted of 139 people: 56 men (40.3%) and 83 women (59.7%), with an average age 53.88 years. The control group consisted of 320 people: 116 men (36.3%) and 204 women (63.7%). The average age of the control group was 55.78 years. The demographic data of the test subjects are listed in Table 2.

An analysis of the genotypes and alleles of *TRAF2* rs867186, *TAB2* rs237025, *IKBKB* rs13278372 in the PA and control groups revealed a statistically significant difference between the *TRAF2* rs867186 genotypes (AA, AG, GG): 79.1%, 17.3%, 3.6% vs. 55.3%, 20.9%, 23.8%, *p* < 0.001, respectively. In addition, the G allele is statistically significantly less frequent in the PA group than in the control group (12.2% vs. 34.2%, *p* < 0.001). No statistically significant differences were found when comparing the distribution of genotypes and alleles of *TAB2* rs237025 and *IKBKB* rs13278372 between the groups (Table 3).

A binary logistic regression analysis was performed to evaluate the influence of *TRAF2* rs867186, *TAB2* rs237025, and *IKBKB* rs13278372 on the occurrence of PAs. We found that the AG genotype of the *TRAF2* rs867186 polymorphism reduced the odds of PA occurrence by 1.74-fold compared to the AA genotype (OR = 0.576; 95% CI: 0.341–0.973; *p* < 0.001), and the GG genotype reduced the odds of PA occurrence by 9.43-fold compared to the AA genotype (OR = 0.106; 95% CI: 0.042–0.270; *p* < 0.001). According to the dominant model, the GG and AG genotypes reduce the odds of PA occurrence by 3.07-fold compared to the AA genotype (OR = 0.326; 95% CI: 0.205–0.519; *p* < 0.001). We also found that the GG genotype, compared to the AA and AG genotypes combined, reduced the odds of PA occurrence by 8.33-fold, according to the recessive model (OR = 0.120; 95% CI: 0.047–0.303; *p* < 0.001). Each G allele reduced the odds of PA occurrence by 2.49-fold according to the additive model (OR = 0.401; 95% CI: 0.286–0.563; *p* < 0.001) (Table 4). The binary logistic regression analysis of *TAB2* rs237025 and *IKBKB* rs13278372 revealed no statistically significant results.

When analyzing the AA, AG, and GG genotypes of the *TRAF2* gene rs867186 polymorphism, statistically significant differences were found between the microadenoma and control groups: 81.3%, 18.8%, 0.0% vs. 55.3%, 20.9%, 23.8%, *p* < 0.001, respectively. In addition, statistically significant results were obtained when comparing the macroadenoma and control groups: 78.0%, 16.5%, 5.5% vs. 55.3%, 20.9%, 23.8%, respectively, *p* < 0.001. It was found that the G allele was statistically significantly less frequent in the microadenoma group than in the control group: 9.4% vs. 34.2%, *p* < 0.001. In addition, the G allele was found to be statistically significantly less frequent in the macroadenoma group than in the control group: 13.7% vs. 34.2, *p* < 0.001. No statistically significant differences were found when analyzing the distribution of genotypes and alleles of the *TAB2* rs237025 and *IKBKB* rs13278372 polymorphisms between the groups (Table 5).

A binary logistic regression analysis was performed to evaluate the influence of *TRAF2* rs867186, *TAB2* rs237025, and *IKBKB* rs13278372 on the occurrence of microadenomas and macroadenomas. We found that the GG and AG genotypes of the *TRAF2* rs867186 polymorphism together reduced the odds of microadenoma occurrence by 3.5-fold compared to the AA genotype according to the dominant model (OR = 0.286; 95% CI: 0.134–0.609; *p* = 0.001). In addition, each G allele was found to reduce the odds of a microadenoma occurring by 3.1-fold according to the additive model (OR = 0.325; 95% CI: 0.176–0.599; *p* < 0.001).

When investigating pituitary macroadenomas, we found that the GG genotype of the *TRAF2* polymorphism rs867186 reduced the odds of macroadenoma occurrence by 6.1-fold compared to the AA genotype according to the codominant model (OR = 0.164; 95% CI: 0.064–0.422; *p* < 0.001). In addition, the GG and AA genotypes together reduce the odds by 2.9-fold compared to the AA genotype (OR = 0.349; 95% CI: 0.203–0.600; *p* < 0.001). According to the recessive model, the GG genotype reduces the odds of the occurrence of a pituitary macroadenoma by 5.3-fold compared to the AA and AG genotypes combined (OR = 0.187; 95% CI: 0.073–0.477; *p* < 0.001). According to the additive model, the G allele reduces the odds by 2.2-fold (OR = 0.447; 95% CI: 0.305–0.656; *p* < 0.001). An analysis of the gene polymorphisms *TAB2* rs237025 and *IKBKB* rs13278372 in patients with pituitary microadenoma or macroadenoma revealed no statistically significant differences (Table 6).

**Figure 1:**
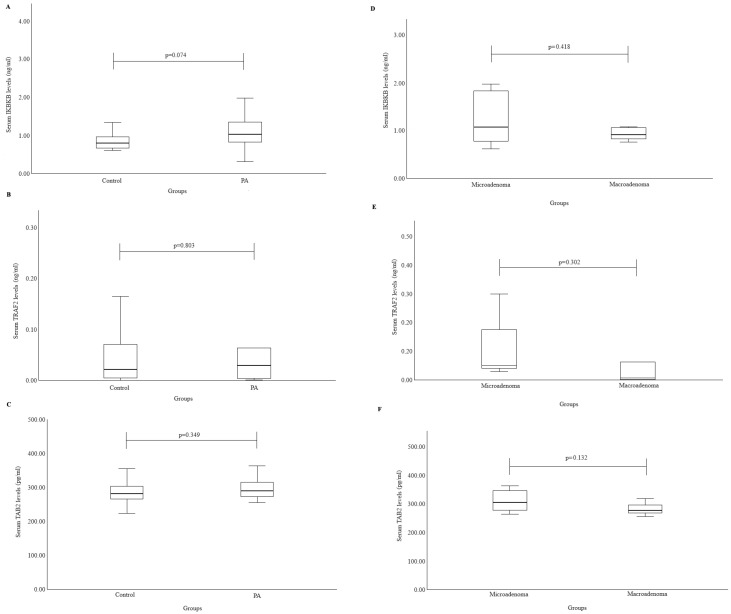
Serum *IKBKB*, *TRAF2*, and *TAB2* levels between groups (Mann–Whitney U test was used). (**A**) Serum *IKBKB* levels (ng/mL) in PA and control groups; (**B**) serum *TRAF2* levels (ng/mL) in PA and control groups; (**C**) serum *TAB2* levels (pg/mL) in PA and reference groups; (**D**) serum *IKBKB* levels (ng/mL) between microadenoma and macroadenoma groups; (**E**) serum *TRAF2* levels (ng/mL) between microadenoma and macroadenoma groups; (**F**) serum *TAB2* levels (pg/mL) between microadenoma and macroadenoma groups.

To evaluate the relationship between the *IKBKB* protein and the manifestation of PAs, we measured the *IKBKB* protein levels in both healthy individuals and PA patients. No statistically significant differences were observed (median (IQR): 0.80 (0.34) vs. 1.03 (0.82), *p* = 0.074). These results are shown in Figure 1A. For *TRAF2* protein levels, we analyzed the healthy and PA patient groups but found no statistically significant differences (median (IQR): 0.02 (0.07) vs. 0.03 (0.18), *p* = 0.803). The results are shown in Figure 1B. Similarly, to assess the relationship between the *TAB2* protein and the manifestation of PAs, we evaluated its concentration in both healthy individuals and PA patients, again finding no statistically significant differences (median (IQR): 281.74 (40.84) vs. 290.50 (45.34), *p* = 0.349). The results are displayed in Figure 1C.

We also analyzed protein concentrations in the microadenoma and macroadenoma groups to evaluate the relationship between *TRAF2*, *IKBKB*, and *TAB2* proteins and PA size. However, no statistically significant differences were found in these comparisons (Figure 1D,E,F, respectively)).

To evaluate the correlation between the Ki-67 labeling index and the size and invasiveness of pituitary adenomas, 76 PA tissue samples were analyzed. The Ki-67 labeling index was assessed in 38 women and 38 men. The results showed no significant difference in the Ki-67 labeling index between women and men (*p* = 0.375) (Table 7). Further immunohistochemical analysis showed no statistical significance in relation to tumor invasiveness (*p* = 0.176) (Table 8) or size (*p* = 0.173) (Table 9).

No statistically significant results were found when analyzing the associations of *TRAF2* rs867186, *TAB2* rs237025, and *IKBKB* rs13278372 polymorphisms with the Ki-67 labeling index (Table 10).

### Summary of Results

This study investigated the association of *TRAF2* rs867186, *TAB2* rs237025, and *IKBKB* rs13278372 polymorphisms with pituitary adenoma (PA) in a case–control study involving 459 subjects (139 PA patients and 320 controls). This study found significant differences in the genotype distribution of the *TRAF2* rs867186 polymorphism between PA patients and controls. Specifically, the G allele was significantly less frequent in PA patients, with a frequency of 12.2% compared to 34.2% in the control group (*p* < 0.001). Additionally, the AG and GG genotypes were associated with a reduced risk of developing a PA, particularly the GG genotype, which had an odds ratio of 0.106 (*p* < 0.001). These findings were consistent across both microadenoma and macroadenoma subgroups.

In contrast, there were no significant differences in the genotype and allele distributions of the *TAB2* rs237025 and *IKBKB* rs13278372 polymorphisms between PA patients and controls, and no significant associations with the occurrence of PAs were found.

Protein expression analysis revealed no significant differences in the levels of *TRAF2*, *IKBKB*, and *TAB2* proteins between PA patients and controls. Similarly, there were no significant differences in protein levels between the microadenoma and macroadenoma groups.

The Ki-67 labeling index showed no significant differences based on gender, tumor size, invasiveness, or any of the polymorphisms studied.

## 4. Discussion

Tumorigenesis in PAs is an incompletely understood process involving the activation of oncogenes, inactivation of tumor suppressor genes, and abnormal growth of pituitary cells [30]. Although whole genome sequencing studies have made significant progress in identifying their pathogenesis, the genetics of a significant proportion of pituitary tumors is still unclear [31].

Our study investigated the relationships between *TRAF2* (rs867186), *TAB2* (rs237025), *IKBKB* (rs13278372) gene polymorphisms and *TRAF2*, *TAB2*, *IKBKB* protein levels with clinical and morphological features of PAs.

Recently, nuclear factor kappa B (NFκB) and its effect on the cell have been a particularly important topic of cancer research. NFκB has been found to play an essential role in regulating immune response, inflammation, cell differentiation, proliferation, and apoptosis [32,33,34]. In addition, there is evidence that NFκB activation is frequently associated with the development of solid and hematopoietic tumors [35,36]. On the other hand, some studies suggest that NFκB may act as a tumor suppressor by directly regulating Fas transcription [37]. Since *TRAF2*, *IKBKB*, and *TAB2* proteins are directly or indirectly involved in NFκB activation, the influence of these proteins on tumorigenesis is discussed in many studies related to cancer occurrence. Still, the genetic factors associated with PAs remain unknown. To our knowledge, this study is the first to examine the associations of *TRAF2* rs867186, *TAB2* rs237025, *IKBKB* rs13278372 polymorphisms and *TRAF2*, *TAB2*, *IKBKB* proteins with clinical and morphological features of PAs and the Ki-67 labeling index.

The *TRAF2* protein plays an important role in cell signaling pathways involving the activation of transcription factors such as NFκB and others [13]. *TRAF2* has been shown to function as an oncogene in breast, gastric, and prostate cancers [38,39,40]. Alterations in *TRAF2* gene expression have also been found in diffuse large B-cell lymphoma and hepatocellular carcinoma [15,16]. In addition, Zhu and colleagues found that *TRAF2* expression significantly increased in nasopharyngeal carcinoma (NPC) cells. Silencing *TRAF2* with short hairpin RNA (shRNA) reduced NPC cell proliferation and colony formation, and its overexpression was linked to high radioresistance [41]. Similarly, Song et al. discovered that *TRAF2* promotes pancreatic cancer development by interacting with Copine 1 (CPNE1). Silencing *CPNE1* with siRNA reduced both CPNE1 and *TRAF2* levels, decreasing pancreatic cancer cell proliferation [42].

The rs867186 polymorphism within the *TRAF2* gene may influence the protein’s functionality and, consequently, the downstream signaling processes. Our findings indicate a significant association between the *TRAF2* rs867186 polymorphism and the incidence of PAs, with the G allele and GG genotype being less frequent among PA patients and associated with reduced odds of developing PAs. There is a notable difference in the distribution of *TRAF2* rs867186 genotypes between PA patients and controls. The lower frequency of the G allele in PA patients suggests a potential protective effect. Further research is warranted to elucidate the precise mechanisms by which this polymorphism influences *TRAF2* function and to explore its utility in clinical practice. However, no data on the association of *TRAF2* with PAs are available so far; however, studies are analyzing *TRAF2* gene polymorphisms with other pathologies.

Gene expression studies performed by Kuehl and colleagues showed that multiple myeloma is associated explicitly with *TRAF2* and *TRAF3* gene mutations that promote activation of the NFκB system via an alternative pathway [14]. In addition, *TRAF2* gene alterations are associated with diffuse large B-cell lymphoma (DLBCL). It has been found that 10 percent of patients with DLBCL have a mutation or deletion in the *TRAF2* gene [15]. *TRAF2* has also been investigated as a prognostic biomarker for prostate cancer. Wei and colleagues found that *TRAF2* expression was statistically significantly (*p* < 0.001) higher in tumorous prostate tissue compared to healthy tissue. In addition, high *TRAF2* expression was statistically significantly (*p* < 0.05) associated with poorer recurrence-free survival [43]. In conclusion, the *TRAF2* protein plays an important role in various biological processes, including cell proliferation, differentiation, and apoptosis. However, increased expression of *TRAF2*, which determines the activation of the NFκB system, is associated with the development of various tumors [44].

The *TAB2* protein is known as a TAK1 activator involved in IL-1-dependent activation of NFκB and MAPK8/JNK [22]. Prolonged activation of TAK1, which the *TAB2* protein can induce, is associated with non-small-cell lung cancer [45]. In addition, the TAK1–*TAB2*–TAB3 signaling axis has been shown to play an important role in carcinoma-induced bone destruction [46]. The *TAB2* protein is also known to promote the activation of EMT and PI3K-AKT signaling pathways associated with tumor cell metastasis and proliferation through indirect activation of NFκB [25]. The influence of *TAB2* gene polymorphisms on the occurrence of neoplastic diseases has not yet been investigated in detail, but the study by Huang and co-authors found that the A allele of the *TAB2* polymorphism rs237028 increases the likelihood of epithelial ovarian tumors by 1.45-fold (GS = 1.45; 95% CI = 1.07–1.96; *p* = 0.016). In addition, the AA genotype of the *TAB2* rs237028 polymorphism increases this probability by 1.66-fold compared to the AG and GG genotypes combined [21].

On the other hand, there are still no data on the association of *TAB2* polymorphisms with PAs. In this study, we analyzed the associations between the *TAB2* rs237025 polymorphism and the *TAB2* protein with the occurrence of PAs, size, and the Ki-67 labeling index, but we found no statistically significant differences. *TAB2* is associated with squamous cell carcinoma of the head and neck. A study by Liu and colleagues found that increased expression of *TAB2* promotes the development of squamous cell carcinoma of the oral cavity by stimulating the proliferation of tumor cells, which is associated with a poor prognosis. On the other hand, low expression of *TAB2* suppresses the uncontrolled proliferation of tumor cells and promotes their apoptosis. Furthermore, *TAB2* has been shown to regulate tumorigenesis via the epithelial–mesenchymal transition (EMT) and the PI3K-AKT pathway [20]. Liu and co-authors found that the deletion of *TAB2* significantly reduced the expression of key genes involved in the EMT and PI3K-AKT signaling pathways. On the other hand, high expression of *TAB2* promotes the expression of the latter genes, suggesting that *TAB2* plays an essential role in the regulation of EMT and PI3K-AKT signaling pathways [20]. *TAB2* gene polymorphisms are known to be associated with malignant epithelial ovarian tumors. Huang et al. found that the *TAB2* polymorphism (rs237028) was statistically significantly (*p* < 0.05) associated with an increased risk of epithelial ovarian tumors, in contrast to the *TAB2* polymorphisms rs521845 and rs652921. In addition, most patients with the rs237028-A allele were older than 50 years and had tumors with a higher degree of differentiation and malignancy (G2 and G3) [14,21].

Our analyzed protein *IKBKB* and *IKBKB* gene polymorphism rs13278372 did not reveal statistically significant associations with PAs.

The IKKβ protein, encoded by the *IKBKB* gene, can activate NFκB through IκB phosphorylation [26]. There are data showing that the IKKβ protein is associated with the development of lung adenocarcinoma, melanoma, pancreatic cancer, and gastric cancer. A study by Xia and colleagues showed that the IKKβ protein promotes the conversion of lung alveolar epithelial cells into tumor cells. On the other hand, chemical inhibition of IKKβ in tumorous lung cells inhibited further cell and tumor growth [47]. Yang and co-authors found that the deletion of the *IKBKB* gene can have a dual effect on melanoma development, depending on the cells involved: in melanocytes, the absence of the IKKβ protein inhibits malignancy, and in myeloid cells, it inhibits phagocytic function, which is important for the destruction of cancer cells [48]. Baumann and colleagues found that the IKKβ protein significantly increases the risk of acute pancreatitis by promoting leukocyte infiltration in pancreatic tissue. At the same time, the deletion of *IKBKB* reduces the risk of pancreatic ductal adenocarcinoma and causes less severe pancreatic lesions [49,50]. In addition, a retrospective study by Gong and colleagues found an association between the *IKBKB* polymorphism rs2272736 and gastric cancer. It was found that patients with the G allele of the *IKBKB* rs2272736 polymorphism survived statistically significantly (*p* < 0.05) longer than patients with the A allele of rs2272736 [29]. On the other hand, there are currently no data on the association of *IKBKB* polymorphisms with PAs. This study analyzed the association between *IKBKB* polymorphism rs13278372 and *IKBKB* proteins with PA expression, size, and Ki-67 labeling index, but no statistically significant differences were found. There are data showing that *IKBKB* polymorphisms are associated with survival in gastric cancer. A retrospective study by Gong and colleagues found that patients with the *IKBKB* rs2272736 G allele survived statistically significantly (*p* < 0.05) longer than those with the rs2272736 A allele. In addition, the risk of dying from gastric cancer was lower in individuals with the AA genotype than in those with the GG and GA genotypes [29].

## 5. Conclusions

This study found that the *TRAF2* rs867186 polymorphism, particularly the AG and GG genotypes, is associated with reduced odds of PA. These findings suggest *TRAF2* rs867186 may reduce PA odds and warrants further research as a genetic marker for PA susceptibility.

## Figures and Tables

**Table 1:** Polymorphism detection protocol.

Polymorphism	RT-PCR Condition Protocol
*TRAF2* (rs867186) *TAB2* (rs237025) *IKBKB* (rs13278372)	95 °C 10 min 45 cycles 92 °C 15 s 60 °C 60 s

**Table 2:** Demographic characteristics.

Characteristics		Group		*p*-Value
		PA Group *n* (%)	Control Group *n* (%)	
Gender	Male	56 (40.3)	116 (36.3)	0.412 ^1^
	Female	83 (59.7)	204 (63.7)	
Age Mean (St. deviation)		53.88 (13.96)	55.78 (17.95)	0.223 ^2^
Invasiveness: Invasive PA/Noninvasive PA		83/54	NA	-
Size: Micro PA/Macro PA		48/91	NA	-
Ki67: <1% 1% >1%		52 10 14	NA	-

^1^ Student *t* test was used; ^2^ Pearson χ^2^ test was used.

**Table 3:** Genotype and allele frequencies of single-nucleotide polymorphisms (*TRAF2* rs867186, *TAB2* rs237025, *IKBKB* rs1327837) within PA and control groups.

Gene, SNP Genotype, Allele	PA Group, *n* (%)	Control Group, *n* (%)	*p*-Value
*TRAF2* rs867186 AA AG GG Total Allele A G	110 (79.1) 24 (17.3) 5 (3.6) 139 (100) 244 (87.8) 34 (12.2)	177 (55.3) 67 (20.9) 76 (23.8) 320 (100) 421 (65.8) 219 (34.2)	<0.001 <0.001
*TAB2* rs237025 GG GA AA Total Allele G A	46 (33.1) 68 (48.9) 25 (18.0) 139 (100) 160 (57.6) 118 (42.4)	100 (31.3) 161 (50.3) 59 (18.4) 320 (100) 361 (56.4) 279 (43.6)	0.927 0.747
*IKBKB* rs13278372 CC AC AA Total Allele C A	111 (79.9) 27 (19.4) 1 (0.7) 139 (100) 249 (89.6) 29 (10.4)	255 (79.7) 60 (18.8) 5 (1.6) 320 (100) 570 (89.1) 70 (10.9)	0.759 0.820

**Table 4:** *TRAF2* rs867186, *TAB2* rs237025, *IKBKB* rs13278372 binary logistic regression analysis within patients with pituitary adenoma and control group subjects.

Model	Genotype/Allele	OR (95% CI)	*p*-Value	AIC
*TRAF2* rs867186				
Codominant	AG vs. AA GG vs. AA	0.576 (0.341–0.973) 0.106 (0.042–0.270)	<0.001 <0.001	528.616
Dominant	GG + AG vs. AA	0.326 (0.205–0.519)	<0.001	540.141
Recessive	GG vs. AA + AG	0.120 (0.047–0.303)	<0.001	531.076
Overdominant	AG vs. AA + GG	0.788 (0.471–1.320)	0.365	564.121
Additive	G	0.401 (0.286–0.563)	<0.001	529.598
*TAB2* rs237025				
Codominant	GA vs. GG AA vs. GG	0.918 (0.586–1.440) 0.921 (0.514–1.651)	0.710 0.783	566.808
Dominant	AA + GA vs. GG	0.919 (0.601–1.406)	0.697	564.808
Recessive	AA vs. GG + GA	0.970 (0.579–1.627)	0.908	564.946
Overdominant	GA vs. GG + AA	0.946 (0.635–1.409)	0.784	564.884
Additive	A	0.954 (0.716–1.270)	0.745	564.853
*IKBKB* rs13278372				
Codominant	AC vs. CC AA vs. CC	1.034 (0.623–1.715) 0.459 (0.053–3.978)	0.898 0.480	566.346
Dominant	AA + AC vs. CC	0.990 (0.603–1.625)	0.967	564.957
Recessive	AA vs. CC + AC	0.457 (0.053–3.944)	0.476	564.362
Overdominant	AC vs. CC + AA	1.045 (0.630–1.732)	0.865	564.930
Additive	A	0.949 (0.603–1.495)	0.822	564.908

**Table 5:** Genotype and allele frequencies of single-nucleotide polymorphisms (*TRAF2* rs867186, *TAB2* rs237025, *IKBKB* rs1327837) within microadenoma, macroadenoma, and control groups.

Gene, SNP Genotype, allele	Control Group, *n* (%)	Microadenoma Group, *n* (%)	*p*-Value	Macroadenoma Group *n* (%)	*p*-Value
*TRAF2* rs867186 AA AG GG Total Allele A G	177 (55.3) 67 (20.9) 76 (23.8) 320 (100) 421 (65.8) 219 (34.2)	39 (81.3) 9 (18.8) 0 (0.0) 48 (100) 87 (90.6) 9 (9.4)	<0.001 <0.001	71 (78.0) 15 (16.5) 5 (5.5) 91(100) 157 (86.3) 25 (13.7)	<0.001 <0.001
*TAB2* rs237025 GG GA AA Total Allele G A	100 (31.3) 161 (50.3) 59 (18.4) 320 (100) 361 (56.4) 279 (43.6)	18 (37.5) 21 (43.8) 9 (18.7) 48 (100) 57 (59.4) 39 (40.6)	0.646 0.584	28 (30.8) 47 (51.6) 16 (17.6) 91 (100) 103 (56.6) 79 (43.4)	0.971 0.964
*IKBKB* rs13278372 CC AC AA Total Allele C A	255 (79.7) 60 (18.8) 5 (1.6) 320 (100) 570 (89.1) 70 (10.9)	40 (83.3) 7 (14.6) 1 (2.1) 48 (100) 87 (90.6) 9 (9.4)	0.765 0.645	71 (78.0) 20 (22.0) 0 (0.0) 91 (100) 162 (89.0) 20 (11.0)	0.401 0.984

**Table 6:** *TRAF2* rs867186, *TAB2* rs237025, *IKBKB* rs13278372 binary logistic regression analysis within patients with microadenoma or macroadenoma and control group subjects.

Model	Genotype/Allele	OR (95% CI)	*p*-Value	AIC
Microadenoma				
*TRAF2* rs867186				
Codominant	AG vs. AA GG vs. AA	0.610 (0.280–1.326) -	0.212 -	263.298
Dominant	GG + AG vs. AA	0.286 (0.134–0.609)	0.001	274.342
Recessive	GG vs. AA + AG	-	-	-
Overdominant	AG vs. AA + GG	0.871 (0.402–1.888)	0.727	286.864
Additive	G	0.325 (0.176–0.599)	<0.001	267.349
*TAB2* rs237025				
Codominant	GA vs. GG AA vs. GG	0.725 (0.368–1.426) 0.847 (0.358–2.008)	0.351 0.707	288.124
Dominant	AA + GA vs. GG	0.758 (0.403–1.423)	0.388	286.257
Recessive	AA vs. GG + GA	1.021 (0.469–2.222)	0.959	286.986
Overdominant	GA vs. GG + AA	0.768 (0.417–1.415)	0.397	286.267
Additive	A	0.884 (0.571–1.371)	0.583	286.685
*IKBKB* rs13278372				
Codominant	AC vs. CC AA vs. CC	0.744 (0.318–1.742) 1.275 (0.145–11.198)	0.495 0.827	286.432
Dominant	AA + AC vs. CC	0.785 (0.153–1.758)	0.556	286.626
Recessive	AA vs. CC + AC	1.340 (0.153–11.726)	0.791	286.923
Overdominant	AC vs. CC + AA	0.740 (0.316–1.730)	0.487	286.478
Additive	A	0.849 (0.416–1.734)	0.653	286.779
Macroadenoma				
*TRAF2* rs867186				
Codominant	AG vs. AA GG vs. AA	0.558 (0.299–1.042) 0.164 (0.064–0.422)	0.067 <0.001	416.570
Dominant	GG + AG vs. AA	0.349 (0.203–0.600)	<0.001	420.363
Recessive	GG vs. AA + AG	0.187 (0.073–0.477)	<0.001	418.170
Overdominant	AG vs. AA + GG	0.745 (0.403–1.380)	0.349	435.671
Additive	G	0.447 (0.305–0.656)	<0.001	415.305
*TAB2* rs237025				
Codominant	GA vs. GG AA vs. GG	1.043 (0.613–1.772) 0.969 (0.484–1.938)	0.887 0.928	438.523
Dominant	AA + GA vs. GG	1.023 (0.618–1.693)	0.930	436.574
Recessive	AA vs. GG + GA	0.944 (0.513–1.735)	0.852	436.547
Overdominant	GA vs. GG + AA	1.055 (0.662–1.681)	0.822	434.531
Additive	A	0.992 (0.708–1.390)	0.964	436.580
*IKBKB* rs13278372				
Codominant	AC vs. CC AA vs. CC	1.197 (0.677–2.118) -	0.536 -	435.686
Dominant	AA + AC vs. CC	1.105 (0.627–1.946)	0.729	434.463
Recessive	AA vs. CC + AC	-	-	-
Overdominant	AC vs. CC + AA	1.221 (0.690–2.159)	0.493	436.121
Additive	A	1.005 (0.594–1.701)	0.984	436.581

**Table 7:** *Ki-67* labeling index considering the gender of pituitary adenoma patients.

Gender	Ki-67 LI			*p*-Value
	<1%	1%	>1%	
Females	25 (48.1%)	7 (70.0%)	6 (42.9%)	0.375
Males	27 (51.9%)	3 (30%)	8 (57.1%)	

**Table 8:** *Ki-67* labeling index considering the size of pituitary adenoma.

Size	Ki-67 LI			*p*-Value
	<1%	1%	>1%	
Micro A *n* = 21 (27.6%)	11 (21.2%)	4 (40.0%)	6 (42.9%)	0.176
Macro PA *n* = 55 (72.4%)	41 (78.8%)	6 (60.0%)	8 (57.1%)	

**Table 9:** *Ki-67* labeling index considering invasiveness of pituitary adenoma.

Invasiveness	Ki-67 LI			*p*-Value
	<1%	1%	>1%	
Noninvasive PA *n* = 24 (33.8%)	20 (40.8%)	2 (20.0%)	2 (16.7%)	0.173
Invasive PA *n* = 47 (66.2%)	29 (59.2%)	8 (80.0%)	10 (83.3%)	

**Table 10:** *TRAF2* rs867186, *TAB2* rs237025, and *IKBKB* rs13278372 associations with Ki-67 labeling index.

Gene, SNP Genotype, Allele	<1% (%)	1% (%)	>1% (%)	*p*-Value
*TRAF2* rs867186 AA AG GG Total Allele A G	39 (75.0) 10 (19.2) 3 (5.8) 52 (100) 88 (84.6) 16 (15.4)	8 (80.0) 2 (20.0) 0 (0.0) 10 (100) 18 (90.0) 2(10.0)	12 (85.7) 2 (14.3) 0 (0.00) 14 (100) 26 (92.9) 2 (7.1)	0.787 0.469
*TAB2* rs237025 GG GA AA Total Allele G A	13 (25.0) 30 (57.7) 9 (17.3) 52 (100) 56 (53.8) 48 (46.2)	7 (70.0) 2 (20.0) 1 (10.0) 10(100) 16 (80.0) 4 (20.0)	5 (35.7) 6 (42.9) 3 (21.4) 14 (100) 16 (57.1) 12 (42.9)	0.084 0.095
*IKBKB* rs13278372 CC AC AA Total Allele C A	42 (80.8) 9 (17.3) 1 (1.9) 52 (100) 93 (89.4) 11 (10.6)	7 (70.0) 3 (30.0) 0 (0.0) 10 (100) 17 (85.0) 3 (15.0)	12 (85.7) 2 (14.3) 0 (0.0) 14 (100) 26 (92.9) 2 (7.1)	0.820 0.682

## Data Availability

The datasets used and analyzed during the current study are available from the corresponding author upon reasonable request.
